# Supplementary material for: Effect of Hemp Seed Oil on Milk Performance, Blood Parameters, Milk Fatty Acid Profile, and Rumen Microbial Population in Milk-Producing Buffalo: Preliminary Study
Source: Animals (Basel). 2025 Feb 11;15(4):514. doi: 10.3390/ani15040514 (PMC11851683; doi:10.3390/ani15040514)
Supplement: Supplementary file 1 [file animals-15-00514-s001.zip › animals-3414956-supplementary.pdf]

Table S1. Effects of HSO supplementation on dry matter intake and average milk yield and composition in milk-producing buffaloes.

Items[1]	Treatment[2]			SEM[3]	*p*-value[4]	Power of tests		
	H0	H1	H2			H0 vs. H1	H0 vs. H2	H2 vs. H3
DMI (kg/d)	11.63	10.19	10.18	0.289	0.06	0.473	0.639	0.050
Milk production								
Milk yield (kg/d)	7.88	7.32	7.72	0.424	0.98	0.079	0.052	0.065
ECM (kg/d)	12.38	11.90	11.93	0.619	0.95	0.061	0.056	0.050
4%FCM (kg/d)	12.36	12.07	12.07	0.629	0.91	0.054	0.053	0.050
Protein (kg/d)	0.31	0.31	0.29	0.018	0.42	0.050	0.234	0.182
Fat (kg/d)	0.57	0.59	0.55	0.031	0.31	0.079	0.076	0.283
TS (kg/d)	1.35	1.36	1.31	0.076	0.69	0.059	0.144	0.217
SNF (kg/d)	0.74	0.73	0.72	0.044	0.85	0.082	0.144	0.073
Lactose (kg/d)	0.37	0.36	0.37	0.023	0.72	0.417	0.050	0.347
Milk content								
Protein (%)	4.41	4.36	4.12	0.088	0.42	0.057	0.230	0.145
Fat (%)	8.01	8.30	7.75	0.196	0.31	0.079	0.069	0.276
TS (%)	19.11	19.16	18.45	0.223	0.69	0.051	0.171	0.202
SNF (%)	10.38	10.28	10.17	0.102	0.85	0.067	0.113	0.065
Lactose (%)	5.22	5.15	5.22	0.030	0.72	0.139	0.050	0.109
Feed efficiency								
Milk yield/DMI (%)	0.69	0.73	0.74	0.039	0.93	0.064	0.071	0.051
ECM/DMI (%)	1.09	1.19	1.15	0.060	0.74	0.091	0.064	0.057

[1] DMI, dry matter intake; ECM, energy-corrected milk; 4%FCM, 4% fat-corrected milk, TS, total solid; SNF, solid not-fat; [2] Diet supplemented with 0 g/d (H0), 100 g/d (H1), and 200 g/d (H2) of HSO. [3] SEM, standard error of the mean. [4] $p \leq 0.05$ was considered statistically significant.

Table S2. Effects of HSO supplementation on rumen fermentation parameters in milk-producing buffaloes.

Item[1]	Treatment[2]			SEM[3]	*p*-value[4]	Power of tests		
	H0	H1	H2			H0 vs. H1	H0 vs. H2	H2 vs. H3
pH	7.04	7.10	7.08	0.034	0.626	0.120	0.068	0.054
Lactate (mmol/L)	0.22	0.25	0.21	0.012	0.193	0.199	0.061	0.219
NH_3-N (mg/100mL)	12.63	12.59	12.59	0.252	0.909	0.050	0.050	0.050
Acetate (mmol/L)	47.13	44.22	43.38	1.480	0.576	0.121	0.158	0.054
Propionate (mmol/L)	9.55	8.71	9.29	0.400	0.647	0.140	0.057	0.071
Butyrate (mmol/L)	7.19	6.62	6.71	0.321	0.831	0.102	0.088	0.051
TVFA (mmol/L)	63.87	59.55	59.39	2.141	0.659	0.126	0.123	0.050
AA/PA (%)	4.95	5.14	4.74	0.101	0.222	0.121	0.132	0.266

[1] TVFA, total volatile fatty acids; AA/PA, acetate/propionate. [2] Diet supplemented with 0 g/d (H0), 100 g/d (H1), and 200 g/d (H2) of HSO. [3] SEM, standard error of the mean. [4] $p \leq 0.05$ was considered statistically significant.

Table S3. High-throughput sequencing quality statistics and Good ' s coverage for bacterial communities.

Sample ID[1]	Clean reads	Effective reads (%)	Good's coverage (%)
H0.1	48,395	94.59	95.60
H0.2	64,675	95.04	96.00
H0.3	38,861	94.81	96.20
H0.4	41,807	95.76	95.70
H0.5	37,734	94.47	95.90
H0.6	55,888	96.06	96.00
H1.1	42,837	96.37	95.80
H1.2	42,672	96.43	95.40
H1.3	29,691	96.30	95.20
H1.4	48,235	93.82	95.60
H1.5	51,895	95.66	95.70
H2.1	39,771	95.51	95.10
H2.2	53,056	95.68	95.40
H2.3	41,098	95.86	95.20
H2.4	44,768	94.35	96.10
H2.5	43,348	94.97	95.10
H2.6	45,031	96.33	96.20
Average	45,280	95.41	95.66
Minimum	29,691	93.82	95.10
Maximum	64,675	96.43	96.20

[1] Diet supplemented with 0 g/d (H0), 100 g/d (H1), and 200 g/d (H2) of HSO; The numbers represent repetitions.
